# Heavy Metal Mixture Exposure and Insulin Resistance in U.S. Adults: The Mediating Role of Systemic Inflammation

**DOI:** 10.1002/jcla.70300

**Published:** 2026-07-01

**Authors:** Cai Zhang, Shidi Lin, Yu Lin

**Affiliations:** ^1^ Department of Health and Kinesiology University of Illinois Urbana‐Champaign Champaign Illinois USA; ^2^ Shirley Ryan AbilityLab Chicago Illinois USA; ^3^ Xiamen Medical College Xiamen Fujian China

**Keywords:** heavy metals, hs‐CRP, inflammation, insulin resistance, mediation analysis, mixture exposure, NHANES

## Abstract

**Introduction:**

Exposure to heavy metals has been implicated in insulin resistance (IR), yet the mechanisms remain unclear. Systemic inflammation may link metal exposure to metabolic outcomes.

**Methods:**

We analyzed 3042 U.S. adults from NHANES 2021–2023. Blood lead, cadmium, mercury, selenium, and manganese were examined in relation to IR (HOMA‐IR), with high‐sensitivity C‐reactive protein (hs‐CRP) as the inflammatory mediator. We used survey‐weighted regression, Baron–Kenny mediation, and weighted quantile sum (WQS) regression for the mixture, stratified by sex; sensitivity analyses additionally adjusted for body mass index (BMI) and smoking.

**Results:**

In primary models, lead, cadmium, and mercury were inversely associated with HOMA‐IR (lead β = −0.259), as was a WQS mixture dominated by these metals, and hs‐CRP significantly mediated several associations. After additional adjustment for BMI and smoking, the inverse single‐metal and mixture associations remained significant but were attenuated by roughly half, indicating substantial adiposity confounding. hs‐CRP continued to mediate a smaller but significant share (roughly 10% to 30%, vs. 25% to 43% before adjustment). The positive manganese association did not survive BMI adjustment and is not robust. Mediation was larger in females and younger adults.

**Conclusions:**

In a nationally representative sample, blood lead, cadmium, and mercury, individually and as a mixture, were inversely associated with insulin resistance, with hs‐CRP mediating a smaller but significant share after adjustment for adiposity. Because the associations are substantially attenuated by BMI and the design is cross‐sectional, reverse causation is plausible. These findings are hypothesis‐generating and motivate prospective studies.

## Graphical abstract

In a nationally representative sample of 3042 U.S. adults from NHANES 2021–2023, blood lead, cadmium, and mercury, both individually and as a weighted quantile sum (WQS) mixture, were inversely associated with insulin resistance assessed by HOMA‐IR. Systemic inflammation, measured by high‐sensitivity C‐reactive protein (hs‐CRP), significantly mediated a modest but meaningful share of these associations (e.g., 24.8% of the lead association overall). The inflammation‐mediated pathway was most pronounced in younger adults and women, suggesting that low‐grade systemic inflammation partially links blood metal exposure to insulin resistance in a demographically patterned way.

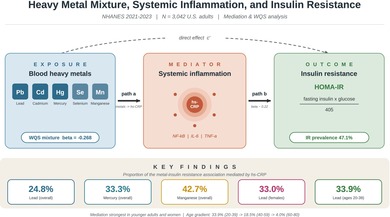

## 1 Introduction

Insulin resistance (IR) is a central feature of type 2 diabetes, metabolic syndrome, and cardiovascular disease, and affects nearly half of U.S. adults [1]. While genetic predisposition, obesity, and sedentary lifestyle are well‐established risk factors, environmental exposures have emerged as important contributors to IR through pathways that are not yet fully understood [2].

Heavy metals are ubiquitous environmental contaminants with established toxicity to metabolic tissues. Lead (Pb), cadmium (Cd), and mercury (Hg) can impair pancreatic beta‐cell function and insulin signaling through oxidative stress, mitochondrial dysfunction, and disruption of calcium homeostasis [3, 4]. Epidemiological studies using NHANES data have reported associations between blood metal concentrations and markers of glucose homeostasis, but results have been inconsistent regarding the direction and magnitude of effects ([5]; [6]). Moreover, most studies have examined individual metals rather than considering simultaneous exposure to multiple metals, which better reflects real‐world conditions.

Systemic inflammation is a biologically plausible pathway linking metal exposure to impaired insulin action, and the individual steps are well characterized. Redox‐active and thiol‐reactive metals, including lead, cadmium, and mercury, generate reactive oxygen species and deplete cellular antioxidant defenses, producing a state of oxidative stress that activates the nuclear factor kappa B (NF‐κB) signaling axis and priming of the NLRP3 inflammasome [7, 8]. NF‐κB activation drives transcription of the pro‐inflammatory cytokines tumor necrosis factor alpha (TNF‐α) and interleukin‐6 (IL‐6), which in turn impair insulin signaling: TNF‐α promotes serine phosphorylation of insulin receptor substrate‐1 (IRS‐1), blunting downstream phosphatidylinositol‐3‐kinase activity and glucose transporter translocation, the molecular lesion that links chronic low‐grade inflammation to insulin resistance [9, 10, 11, 12]. High‐sensitivity C‐reactive protein (hs‐CRP), a hepatic acute‐phase reactant synthesized in response to IL‐6, integrates this cytokine signal into a single, stable, and clinically standardized read‐out of systemic inflammation. We selected hs‐CRP as the mediator because it is the most widely measured inflammatory biomarker in clinical laboratories and because prospective cohort data show that baseline hs‐CRP and IL‐6 predict incident insulin resistance and type 2 diabetes independent of established risk factors, establishing hs‐CRP as a forward‐looking marker of inflammatory metabolic risk rather than a nonspecific correlate [13, 14, 15]. Human population studies support both ends of this pathway for the metals examined here: Combined lead, cadmium, and mercury exposure has been associated with elevated systemic inflammation [16, 17], and insulin resistance itself is a downstream, mechanistically grounded outcome with broad cardiometabolic consequences [18]. If inflammation mediates the metal–IR association, hs‐CRP would mark a measurable and potentially modifiable step on that pathway.

Recent methodological advances enable formal testing of mediation hypotheses and evaluation of chemical mixture effects. The Baron‐Kenny causal steps approach, combined with bootstrap confidence intervals for the indirect effect, provides a rigorous framework for mediation analysis [19]. Weighted quantile sum (WQS) regression allows estimation of the joint effect of correlated metal exposures while identifying the relative contribution of each metal to the mixture [20]. To date, only one study has applied mediation analysis to the metal–inflammation–IR pathway using NHANES data, but it used older survey cycles (2011–2016) and did not evaluate sex‐specific effects or age‐dependent patterns [21].

The present study used data from the most recent NHANES cycle (2021–2023) to (1) examine the associations between five blood metals and HOMA‐IR in U.S. adults, stratified by sex; (2) test whether hs‐CRP mediates these associations using bootstrap‐based mediation analysis; (3) evaluate the joint effect of the metal mixture using WQS regression; and (4) assess whether mediation patterns vary by age group and race/ethnicity.

## 2 Methods

### 2.1 Study Population

Data were obtained from NHANES 2021–2023 (August 2021–August 2023), a nationally representative cross‐sectional survey of noninstitutionalized U.S. civilians conducted by the CDC. The study protocol was approved by the NCHS Research Ethics Review Board (Protocol #2018–01), and all participants provided written informed consent.

**FIGURE 1:**
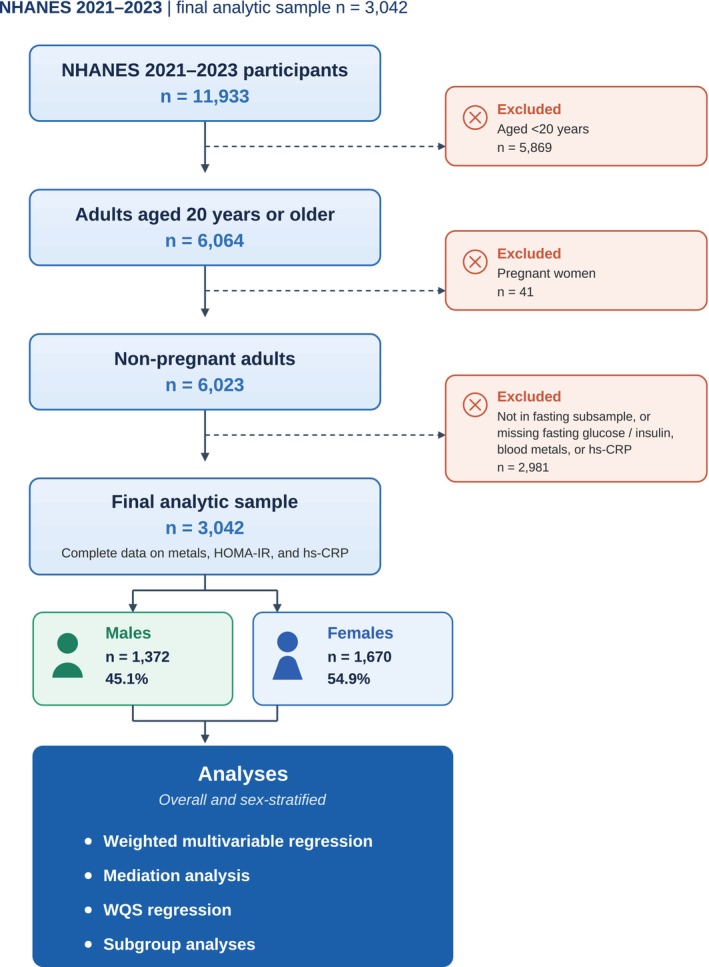
Participant selection flowchart for the NHANES 2021–2023 analytic sample NHANES, National Health and Nutrition Examination Survey; IR, insulin resistance. The final analytic sample included 3,042 adults aged ≥ 20 years, including 1,372 males and 1,670 females. IR prevalence was 51.7% in males and 43.2% in females.

From 11,933 participants, we restricted to adults aged 20 years and older (*n* = 6,064), excluded pregnant women (*n* = 41), and required participants from the fasting insulin subsample with complete data on all five metals, fasting glucose, fasting insulin, and hs‐CRP. The final analytic sample comprised 3042 participants (1372 males, 1670 females). The participant selection process is shown in Figure 1.

### 2.2 Exposure Assessment

Whole blood concentrations of lead, cadmium, total mercury, selenium, and manganese were measured by inductively coupled plasma dynamic reaction cell mass spectrometry (ICP‐DRC‐MS) at the CDC Division of Laboratory Sciences. Detection rates were 100% for lead, 95.7% for cadmium, 74.0% for mercury, and 100% for selenium and manganese. Values below the limit of detection were imputed as LOD/√2 [22]. All metal concentrations were natural log‐transformed for analysis.

### 2.3 Outcome Assessment

Fasting insulin (μU/mL) was measured by a two‐site immunoenzymatically sandwich assay, and fasting plasma glucose (mg/dL) by the hexokinase enzymatic method, both on the fasting morning subsample. The homeostasis model assessment of insulin resistance (HOMA‐IR) was calculated as fasting insulin × fasting glucose / 405 [23]. IR was defined as HOMA‐IR ≥ 2.5, consistent with commonly used clinical thresholds. HOMA‐IR was natural log‐transformed for regression analyses.

### 2.4 Mediator: Hs‐CRP

Serum hs‐CRP (mg/L) was measured by latex‐enhanced nephelometry on the Siemens ProSpec instrument. hs‐CRP is an established biomarker of systemic low‐grade inflammation and has been consistently linked to IR and metabolic syndrome [24]. hs‐CRP was natural log‐transformed for analysis.

### 2.5 Covariates

Covariates were selected a priori using a directed acyclic graph (DAG) that encoded our assumptions about the metal–inflammation–insulin resistance system. The primary model adjusted for variables that are plausible common causes of both blood metal concentration and insulin resistance and that are not themselves on the hypothesized causal pathway: Age (continuous), race and ethnicity (Mexican American, other Hispanic, non‐Hispanic White, non‐Hispanic Black, non‐Hispanic Asian, other or multiracial), serum creatinine (mg/dL, a marker of renal handling and of urinary dilution that affects metal clearance), total cholesterol (mg/dL), triglycerides (mg/dL), and serum 25‐hydroxyvitamin D (nmol/L). We deliberately did not adjust the primary model for body mass index (BMI). Under the reverse‐causation and hemodilution framework that we and others have proposed for the inverse metal–IR associations [25, 26], adiposity plausibly lies on the reverse causal path, in which insulin resistance promotes weight gain, expanded adipose mass increases the volume of distribution, and circulating metal concentrations are thereby diluted. On that causal structure, BMI is a mediator or collider for the metal–IR association rather than a confounder, so conditioning on it in the primary model would induce over‐adjustment bias and could attenuate or distort the very association of interest. Because the correct causal status of BMI is not known with certainty, and because BMI is unambiguously a confounder under the competing hypothesis that metals act through an adiposity‐independent inflammatory pathway, we evaluated BMI in a prespecified sensitivity series rather than excluding it from consideration (Section 2.6 and Section 3.6). Lifestyle and socioeconomic factors that the primary model omitted, namely smoking status, alcohol use, physical activity, dietary energy intake, educational attainment, and the family income‐to‐poverty ratio, were likewise reserved for sensitivity analyses, because each is a candidate confounder of the metal–IR association, but several also lie partly downstream of the exposures or the outcome.

### 2.6 Statistical Analysis

#### 2.6.1 Weighted Multivariable Regression

**FIGURE 2:**
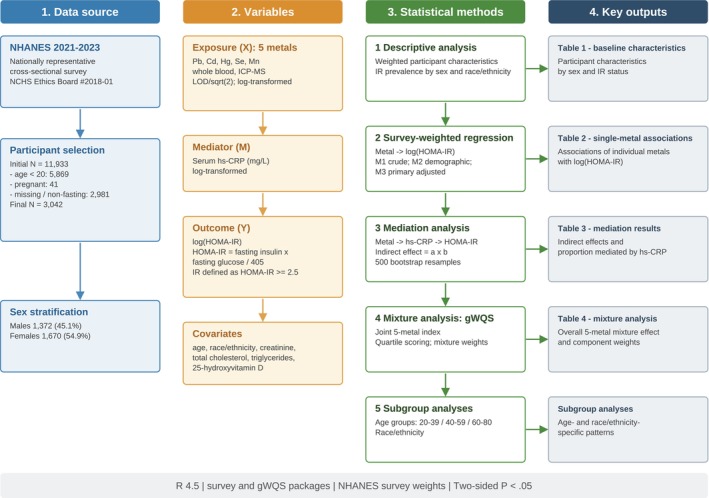
Analytical workflow for evaluating blood metals, inflammation, and insulin resistance in NHANES 2021–2023. The workflow summarizes participant selection, variable definition, and statistical analyses, including survey‐weighted regression, mediation analysis, WQS mixture modeling, and subgroup analyses.

We fitted three sequential survey‐weighted linear regression models relating each log‐transformed metal to log(HOMA‐IR): A crude model (Model 1), a partially adjusted model including age and race and ethnicity (Model 2), and a fully adjusted primary model (Model 3) additionally controlling for creatinine, total cholesterol, triglycerides, and vitamin D, as defined in Section 2.5 (Figure 2). NHANES examination sample weights (WTMEC2YR), sampling strata, and primary sampling units were incorporated using Taylor‐series linearization to account for the complex survey design and to obtain nationally representative estimates. Confidence intervals and *p*‐values were computed on the survey design degrees of freedom (15 for the 2021 to 2023 cycle, reflecting approximately 30 primary sampling units within 15 strata), which yields appropriately conservative inference for a design of this size. We note that the fasting biomarkers (insulin, glucose) are formally associated with the fasting subsample weight (WTSAF2YR); we used the examination weight WTMEC2YR for consistency with the design of the parent analysis, and we address this choice as a limitation (Section 4). Analyses for this revision were executed in R (survey, gWQS); estimates are materially identical to the original submission, with negligible differences (analytic N within 0.6%, coefficients within 0.01) reflecting re‐derivation and complete‐case handling.

#### 2.6.2 Mediation Analysis

**FIGURE 3:**
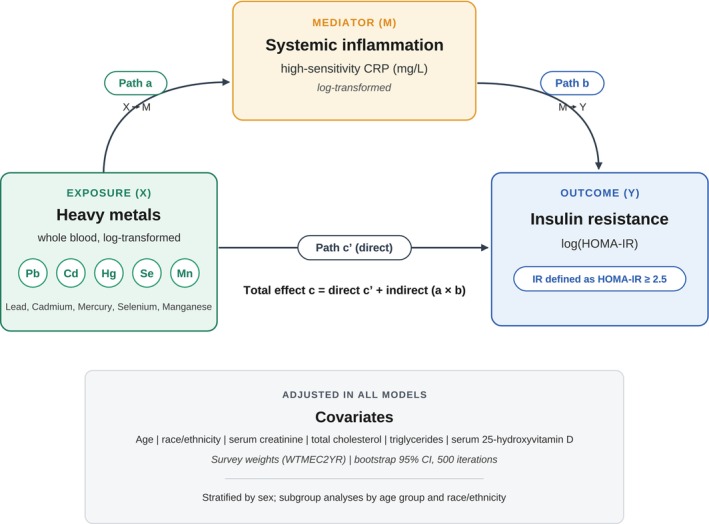
Conceptual mediation framework linking blood metal exposure, systemic inflammation, and insulin resistance. Blood metals were modeled as log‐transformed exposures, hs‐CRP as the mediator, and log(HOMA‐IR) as the outcome. The total effect was decomposed into direct and indirect effects through systemic inflammation. Models were survey‐weighted and adjusted for key demographic and clinical covariates.

We evaluated whether systemic inflammation mediated the associations between metal exposures and insulin resistance using the Baron–Kenny framework (Figure 3). The total effect (path c) of each metal on log (HOMA‐IR) was decomposed into a direct effect (path c') and an indirect effect through log‐transformed hs‐CRP (paths a × b). The proportion mediated was calculated as (a × b) / c × 100%. Statistical significance of the indirect effect was assessed using bootstrap‐derived 95% confidence intervals (500 bootstrap resamples). All mediation models were based on the fully adjusted covariate set to ensure consistency across analyses.

#### 2.6.3 Weighted Quantile Sum (WQS) Regression

To assess the joint effect of correlated metal exposures, we applied weighted quantile sum (WQS) regression using the standard gWQS optimizer [20], with each metal scored in quartiles and the weighted index estimated under a single directional (negative) constraint on a 40% training and 60% validation split with 100 bootstrap ensembles. The WQS coefficient represents the overall mixture effect on log(HOMA‐IR), and the estimated weights, constrained to be non‐negative and to sum to 1, indicate the relative contribution of each metal to the mixture. Mixture models were adjusted for the same covariates as the corresponding single‐metal model, and the mixture was re‐estimated under the BMI plus smoking and full lifestyle adjustment sets described above (Section 3.6).

#### 2.6.4 Subgroup Analyses

To evaluate potential heterogeneity, mediation analyses were repeated across predefined subgroups, including age (20–39, 40–59, and 60–80 years) and race/ethnicity categories. These analyses were exploratory and aimed to assess whether the magnitude of mediation varied across demographic strata.

Sensitivity analyses. To quantify the influence of adiposity and lifestyle factors omitted from the primary model, we refitted the metal–IR associations on a common complete‐case sample (*n* = 2,968) in a nested series: M0 reproduced the primary covariate set; M1 added BMI (continuous); M2 added smoking status (never, former, current); M3 added both BMI and smoking; and M4 (*n* = 1,918) further added alcohol use, physical activity, educational attainment, the family income‐to‐poverty ratio, and total dietary energy intake. M3 was prespecified as the headline robustness model, with M4 reported as supporting evidence given its reduced sample. We repeated the WQS mixture analysis and the hs‐CRP mediation analysis under the M3 and M4 adjustment sets to determine whether the inverse mixture and the inflammatory mediation persisted after accounting for adiposity. Finally, to address whether cardiovascular disease (CVD) could generate spurious metal–IR associations, we estimated the survey‐weighted prevalence of prevalent CVD (self‐reported congestive heart failure, coronary heart disease, angina, myocardial infarction, or stroke) by insulin‐resistance status and added prevalent CVD as a covariate to the primary model. Because the 2021 to 2023 physical‐activity questionnaire (PAQ) carries only recreational‐activity items, M4 adjusted for sedentary time and an indicator of any recreational activity rather than a full metabolic‐equivalent score. Because this nested series is fit on a common complete‐case subsample (*n* = 2,968), its baseline model (M0) differs slightly from the primary Table 1 estimates, which use the full analytic sample.

**TABLE 1:** Associations of log‐transformed metals with log (HOMA‐IR): Fully adjusted weighted regression.

Metal	Males	Females	Overall
Lead	−0.297 (−0.381, −0.214)*	−0.220 (−0.276, −0.141)*	−0.259 (−0.301, −0.204)*
Cadmium	−0.200 (−0.283, −0.121)*	−0.104 (−0.154, −0.047)*	−0.143 (−0.190, −0.102)*
Mercury	−0.102 (−0.140, −0.057)*	−0.068 (−0.105, −0.036)*	−0.085 (−0.122, −0.052)*
Selenium	0.493 (0.094, 0.791)*	0.279 (−0.021, 0.551)	0.401 (0.208, 0.627)*
Manganese	0.328 (0.174, 0.464)*	0.178 (0.080, 0.293)*	0.232 (0.125, 0.334)*

*Note:* beta (95% CI).

* *p* < 0.05. Adjusted for age, race/ethnicity, creatinine, cholesterol, triglycerides, and vitamin D.

#### 2.6.5 Handling of Missing Data

Participants with missing data on any of the primary exposures (metals), outcome (HOMA‐IR), mediator (hs‐CRP), or covariates were excluded from the analytic sample, resulting in a complete‐case analysis. The proportion of missingness for individual variables was low to moderate, and exclusions were primarily driven by the fasting subsample requirement inherent to NHANES. Given the cross‐sectional design and the requirement for complete data across multiple components (laboratory, questionnaire, and examination data), complete‐case analysis was considered appropriate.

To assess the potential impact of missing data, we compared key demographic characteristics between included and excluded participants and found no substantial differences (data not shown), suggesting that selection bias due to missingness was unlikely to materially affect the results.

## 3 Results

### 3.1 Participant Characteristics

The final sample included 3042 adults (45.1% male). The prevalence of IR was 47.1% overall, higher in males (51.7%) than in females (43.2%). Participants with IR had significantly higher hs‐CRP (median 2.89 vs. 1.25 mg/L, *p* < 0.001), triglycerides (122.5 vs. 86.0 mg/dL, *p* < 0.001), and fasting glucose (105 vs. 93 mg/dL, *p* < 0.001) compared with those without IR. Paradoxically, blood lead (0.72 vs. 0.78 μg/dL), cadmium (0.25 vs. 0.29 μg/L), and mercury (0.51 vs. 0.70 μg/L) were lower in the IR group (all *p* < 0.001). IR prevalence varied by race/ethnicity, being highest in Mexican Americans (59.0%) and lowest in non‐Hispanic Asians (41.1%) (Table 2; Table S1).

**TABLE 2:** Participant characteristics by insulin resistance status, NHANES 2021–2023 (*n* = 3,042).

	Normal (*n* = 1,610)	IR (*n* = 1,432)	*p*
Male, *n* (%)	662 (41.1)	710 (49.6)	< 0.001
Age, years	57.0 (39–67)	59.0 (43–68)	0.002
Blood Lead, μg/dL	0.78 (0.51–1.24)	0.72 (0.46–1.07)	< 0.001
Blood Cadmium, μg/L	0.29 (0.18–0.47)	0.25 (0.16–0.42)	< 0.001
Total Mercury, μg/L	0.70 (0.27–1.63)	0.51 (0.23–1.15)	< 0.001
Selenium, μg/L	176.3 (162.7–191.7)	179.6 (165.5–195.0)	< 0.001
Manganese, μg/L	8.68 (7.00–10.76)	9.04 (7.42–11.21)	< 0.001
hs‐CRP, mg/L	1.25 (0.60–2.81)	2.89 (1.27–5.97)	< 0.001
Fasting glucose, mg/dL	93.0 (87–99)	105.0 (97–122)	< 0.001
Fasting insulin, μU/mL	6.06 (4.40–7.96)	16.14 (12.56–23.47)	< 0.001
HOMA‐IR	1.42 (1.00–1.87)	4.31 (3.21–6.61)	< 0.001
Triglycerides, mg/dL	86.0 (65–114)	122.5 (92–171)	< 0.001
Vitamin D, nmol/L	81.4 (61.8–105.0)	70.9 (50.4–96.5)	< 0.001

*Note:* Values are median (IQR) unless otherwise noted. IR = insulin resistance (HOMA‐IR > = 2.5). P from the Mann–Whitney U test.

### 3.2 Metal–HOMA‐IR Associations

In fully adjusted models (Table 1; Table S2), lead, cadmium, and mercury were all inversely associated with log (HOMA‐IR) in both sexes. The associations were strongest for lead (males: β = −0.297; females: β = −0.220; both *p* < 0.05) and remained robust across all three model specifications. In the primary models, selenium and manganese were positively associated with HOMA‐IR (selenium β = 0.493 in males; manganese β = 0.328 in males and 0.178 in females; all *p* < 0.05); as shown in Section 3.6, the manganese association did not persist after adjustment for BMI and is therefore not interpreted as a robust signal. Associations were generally stronger in males than in females. Correlations among the five metals, hs‐CRP, and glycemic biomarkers are shown in Table S3 (visualized as a heatmap in Figure S4), and a forest plot of fully adjusted β estimates by sex is shown in Figure S1.

### 3.3 Mediation by Hs‐CRP

Mediation analysis revealed that hs‐CRP significantly mediated the associations of lead, mercury, and manganese with HOMA‐IR (Table 3). For lead, the indirect effect through hs‐CRP was significant overall (indirect = −0.063, 24.8% mediated, *p* < 0.05) and in both sexes (males: Indirect = −0.035, 11.9% mediated; females: Indirect = −0.071, 33.0% mediated; both *p* < 0.05). The mediation proportion was larger in females than in males for all metals. Mercury showed a consistent mediation pattern (overall: Indirect = −0.029, 33.3% mediated, *p* < 0.05), with the indirect effect driven by mercury's negative association with hs‐CRP (path a) and hs‐CRP's positive association with HOMA‐IR (path b approximately 0.22; the single value 0.381 printed in the originally submitted Table S6 was a transcription error and has been corrected). Manganese showed a positive indirect effect (overall: Indirect = +0.099, 42.7% mediated, *p* < 0.05), consistent with manganese increasing both hs‐CRP and HOMA‐IR. The cadmium–hs‐CRP–IR pathway was significant only in females (overall: Indirect = +0.009, not significant; females: 18.7% mediated, *p* < 0.05); Figure S3 and Table S6 display the corresponding path‐level decompositions by metal and group.

**TABLE 3:** Mediation of metal‐HOMA‐IR associations by hs‐CRP (fully adjusted).

Metal	Sex	Total (c)	Indirect (ab)	95% CI	% Med	Sig
Lead	M	−0.290	−0.035	(−0.052, −0.017)	11.9%	*
	F	−0.217	−0.071	(−0.094, −0.050)	33.0%	*
	All	−0.254	−0.063	(−0.079, −0.050)	24.8%	*
Cadmium	M	−0.192	+0.021	(−0.008, +0.046)	−10.8%	
	F	−0.102	−0.019	(−0.037, −0.004)	18.7%	*
	All	−0.136	+0.009	(−0.007, +0.022)	−6.6%	
Mercury	M	−0.102	−0.031	(−0.040, −0.021)	30.4%	*
	F	−0.068	−0.021	(−0.031, −0.011)	31.4%	*
	All	−0.086	−0.029	(−0.035, −0.021)	33.3%	*
Manganese	M	+0.327	+0.088	(+0.044, +0.134)	26.9%	*
	F	+0.182	+0.077	(+0.055, +0.100)	42.0%	*
	All	+0.231	+0.099	(+0.080, +0.121)	42.7%	*

*Note:* Baron‐Kenny mediation. Indirect = a × b. % Med = indirect/total × 100. * = bootstrap 95% CI excludes zero. M = male, F = female.

### 3.4 Metal Mixture Effects

The overall metal mixture was inversely associated with HOMA‐IR (WQS beta = −0.268; 95% CI −0.311, −0.225; Table 4), with lead, cadmium, and mercury carrying essentially all of the index weight (0.407, 0.325, and 0.268, respectively) and selenium and manganese contributing negligibly (weights approximately 0). The inverse mixture was evident in both sexes (males −0.309; females −0.192; Figure S2).

**TABLE 4:** WQS mixture analysis: Metal weights and mixture coefficients for HOMA‐IR.

	Males	Females	Overall
Mixture beta (95% CI)	−0.309 (−0.375, −0.243)*	−0.192 (−0.245, −0.139)*	−0.268 (−0.311, −0.225)*
Lead weight	0.345	0.399	0.407
Cadmium weight	0.360	0.275	0.325
Mercury weight	0.294	0.323	0.268
Selenium weight	0.000	0.003	0.000
Manganese weight	0.000	0.000	0.000

*Note:* Adjusted for age, race/ethnicity, creatinine, total cholesterol, triglycerides, and vitamin D. Weighted quantile sum regression (gWQS package, directional negative constraint); weights are non‐negative and sum to 1.

* Bootstrap 95% CI excludes zero.

### 3.5 Subgroup Analyses

**FIGURE 4:**
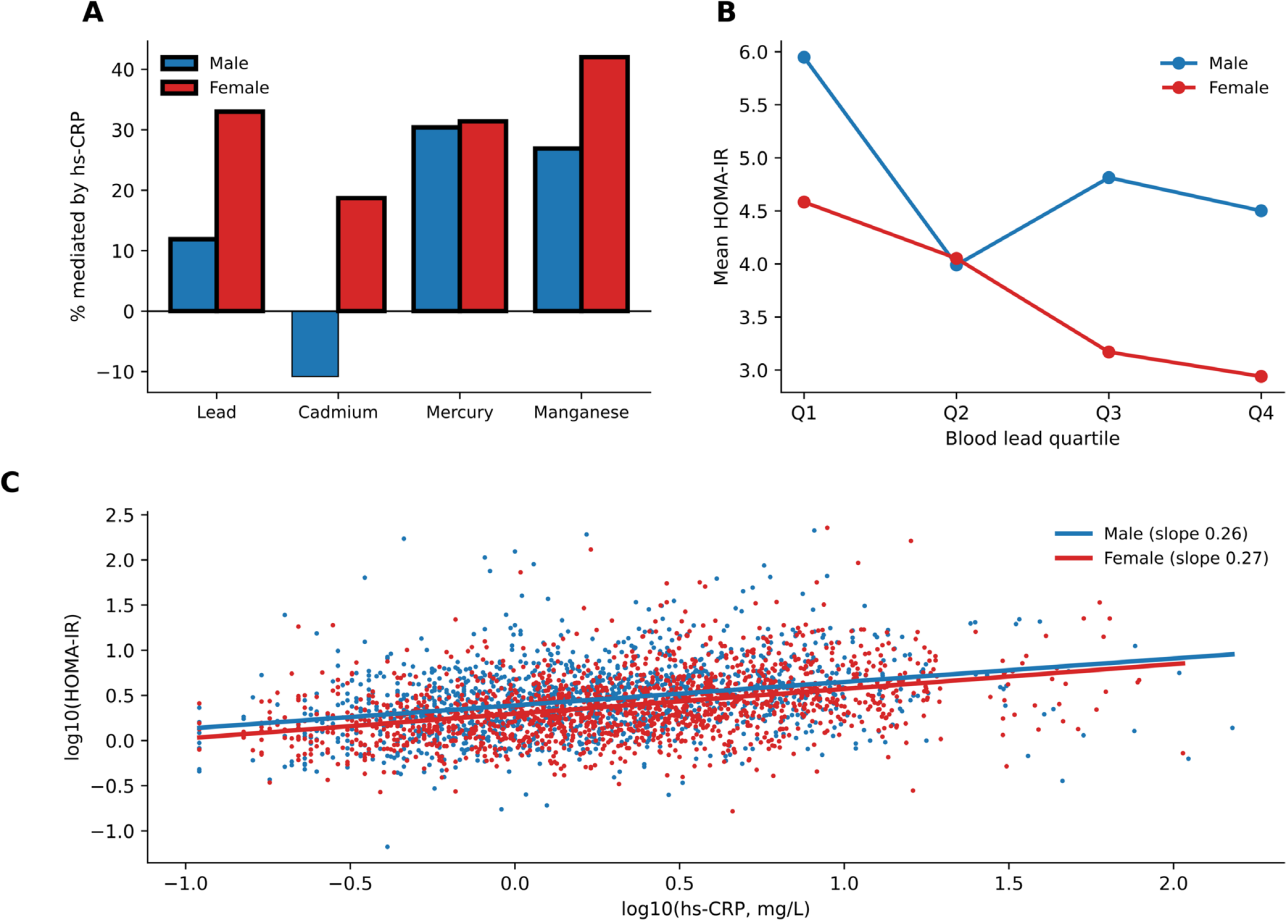
Sex‐specific patterns linking metals, inflammation, and insulin resistance. Panel A shows the proportion of the metal–HOMA‐IR association mediated by hs‐CRP. Black‐outlined bars indicate bootstrap *P* < 0.05. Panel B shows mean HOMA‐IR by lead quartile and sex. Panel C shows log10(hs‐CRP) vs. log10(HOMA‐IR) with fitted trend lines by sex.

The inflammation‐mediated pathway varied substantially by age (Figure 4). The proportion of the lead–IR association mediated by hs‐CRP was 33.9% in adults aged 20–39 years (*p* < 0.05), 18.5% in those aged 40–59 (*p* < 0.05), and only 4.0% in those aged 60–80 (nonsignificant). This age gradient suggests that the inflammatory mechanism is most relevant in younger adults (Table S4; Figure S5).

Race‐stratified analysis showed that the lead–IR association was significant in non‐Hispanic Whites (β = −0.281, *p* < 0.05), non‐Hispanic Blacks (β = −0.327, *p* < 0.05), and Mexican Americans (β = −0.228, *p* < 0.05), but not in non‐Hispanic Asians (β = −0.127, *p* = 00.19), likely due to limited sample size (*n* = 168).

### 3.6 Sensitivity Analyses

We assessed the robustness of the primary associations to adiposity, smoking, and a broader set of lifestyle and socioeconomic covariates on a common complete‐case sample (Table S5; Figure S6). Adding smoking alone (M2) barely changed any estimate (the lead coefficient moved by under 2%, from −0.251 to −0.247). In contrast, adding BMI alone (M1) attenuated the toxic‐metal associations by roughly half to two‐thirds: Lead by 63% (−0.251 to −0.094), cadmium by 47% (−0.136 to −0.072), mercury by 60% (−0.088 to −0.035), and manganese by 74% (+0.228 to +0.059). BMI, rather than smoking, was therefore the operative variable behind the attenuation.

In the combined model adjusting for both BMI and smoking (M3, *n* = 2,968), the inverse associations for lead (−0.093; 95% CI −0.149, −0.037), cadmium (−0.088; 95% CI −0.133, −0.043), and mercury (−0.036; 95% CI −0.066, −0.006) all remained statistically significant, although markedly reduced in magnitude. The positive manganese association did not survive this adjustment (+0.060; 95% CI −0.018, +0.137; *p* = 0.12) and is consequently not interpreted as a robust finding. In the full lifestyle model (M4, *n* = 1,918), which further adjusted for alcohol use, physical activity, education, the income‐to‐poverty ratio, and dietary energy, lead (−0.078; 95% CI −0.141, −0.014) and cadmium (−0.065; 95% CI −0.110, −0.021) remained significant, whereas mercury fell to the null (−0.010; 95% CI −0.048, +0.027). Of the three toxic metals, lead and cadmium were thus the most robust signals and mercury the least. In sex‐stratified sensitivity models, lead remained significant under M4 in males (−0.099; 95% CI −0.183, −0.015) but was attenuated to non‐significance in females (−0.072; 95% CI −0.176, +0.033), a pattern consistent in part with the reduced power of the M4 subsample.

The WQS metal mixture showed the same pattern (Table S7). The inverse mixture index was −0.268 (95% CI −0.311, −0.225) in the baseline model, −0.126 (95% CI −0.167, −0.085) under M3, and −0.085 (95% CI −0.132, −0.039) under M4; all three estimates were statistically significant. Lead, cadmium, and mercury continued to carry essentially all of the index weight (e.g., 0.32, 0.44, and 0.23, respectively, under M3), while selenium and manganese contributed negligibly. The inverse mixture association was therefore attenuated by approximately half after adjustment for adiposity but remained robust in direction and significance.

Mediation by hs‐CRP persisted but shrank substantially after adjustment (Table S8). For lead, the proportion mediated fell from 24.8% to 9.5%; for mercury, from 33.3% to 10.5%; and for manganese, from 42.7% to 30.0%. The hs‐CRP‐to‐HOMA‐IR path coefficient itself fell from about 0.22 to about 0.08 once BMI entered the model, consistent with adiposity acting as a common cause of both systemic inflammation and insulin resistance. The indirect (hs‐CRP‐mediated) effects remained statistically significant in the adjusted models, so the qualitative inference that inflammation lies on the pathway is preserved, but a meaningful share of what the primary models attributed to inflammation is attributable to adiposity, and the magnitude of mediation is correspondingly smaller than the primary estimates imply. Because the manganese total association did not survive adjustment for adiposity, its mediated fraction is reported for completeness and is not interpreted as evidence of a robust pathway.

Prevalent cardiovascular disease was about twice as common among insulin‐resistant participants (12.9%; 95% CI 11.2, 14.6) as among non‐resistant participants (7.0%; 95% CI 5.5, 8.4), against an overall weighted prevalence of 9.8% (95% CI 8.7, 10.9; design‐based *p* < 0.001) (Table S9). Adjusting the primary model for prevalent CVD, however, changed every metal–IR estimate negligibly (all changes ≤ 0.007 on the log scale;e.g., lead −0.254 to −0.253, cadmium −0.136 to −0.143). Cardiovascular disease, therefore, co‐occurs with insulin resistance, as expected clinically, but does not act as a confounder of the metal–IR associations in these data.

## 4 Discussion

This study examined the associations between blood heavy metals and insulin resistance in a nationally representative sample of U.S. adults and formally tested whether systemic inflammation mediates these associations. Three key findings emerged. First, lead, cadmium, and mercury were inversely associated with HOMA‐IR, while selenium and manganese were positively associated. Second, hs‐CRP significantly mediated the metal–IR associations, with mediation proportions ranging from 12% to 42% depending on the metal and sex. Third, the inflammation‐mediated pathway was age‐dependent, being strongest in younger adults and diminishing with age.

The inverse associations between toxic metals (Pb, Cd, Hg) and HOMA‐IR may appear counterintuitive. However, this finding is consistent with several NHANES‐based studies. Wu et al. (2026) reported negative associations between lead and fasting insulin using NHANES 2011–2018, and Menke et al. [5] found similar patterns for cadmium. Several non‐causal explanations may account for these findings. First, reverse causation through adiposity‐driven hemodilution is the most plausible explanation. Insulin‐resistant adults tend to carry more adipose tissue and a larger plasma volume, which dilutes circulating concentrations of blood‐borne metals; the same physiological mechanism has been shown to dilute other circulating biomarkers in obese individuals [27]. Direct support comes from population studies in which adiposity tracks with lower blood metal levels: Blood cadmium, copper, and lead were inversely associated with overweight and obesity, and a multi‐metal mixture was inversely associated with obesity, in a cross‐sectional study using the same blood‐metal and mixture framework as ours [26], echoing the earlier report of Padilla et al. [25]. Our own sensitivity analyses reinforce this interpretation: Adjusting for BMI attenuated the inverse lead, cadmium, and mercury associations by roughly half to two‐thirds, whereas adjusting for smoking alone changed them negligibly (Section 3.6). An attenuation of this size on conditioning for adiposity is exactly what the hemodilution and reverse‐causation account predicts, although, because BMI may be either a confounder or a collider on this pathway, the sensitivity estimates bound rather than resolve the true association. Second, residual confounding by smoking remains possible because smoking raises both metal exposure and metabolic rate [28], even though measured smoking status did not materially change the estimates here. Third, low‐dose metals may transiently stimulate pancreatic beta‐cell insulin secretion through hormetic effects [4]. None of these mechanisms can be distinguished in a cross‐sectional design, and the inverse associations should be read as correlational rather than causal.

In the primary models, hs‐CRP mediated roughly one quarter to two fifths of the metal–IR associations, extending the work of Mao et al. [21], who reported significant inflammatory mediation using NHANES 2011–2016 data. This magnitude, however, is sensitive to adiposity. When BMI and smoking were added (Section 3.6), the proportion mediated fell to roughly 10% for lead and mercury and to about 30% for manganese, and the hs‐CRP‐to‐insulin‐resistance path coefficient fell from about 0.22 to about 0.08 because adiposity is a shared cause of both systemic inflammation and insulin resistance. We therefore interpret hs‐CRP as mediating a smaller but still statistically significant share of the metal–IR associations after accounting for adiposity, rather than the larger share implied by the primary models. The qualitative conclusion that inflammation lies on the pathway is preserved; its quantitative weight is more modest than an analysis omitting BMI would suggest. Within this softened interpretation, our study still adds three contributions beyond Mao et al. [21]: Replication in the most recent NHANES cycle (2021–2023); demonstration of sex‐specific mediation, with larger proportions mediated in females than males; and identification of age‐dependent patterns. The stronger mediation in younger adults may reflect a more responsive inflammatory system with less background inflammation, allowing metal‐induced inflammatory signals to exert a proportionally larger effect on insulin signaling. In older adults, the high prevalence of chronic low‐grade inflammation from multiple sources may attenuate the relative contribution of metal‐induced inflammation.

Our decision to analyze the 2021 to 2023 NHANES cycle alone, rather than pooling multiple cycles, was deliberate. The August 2021 to August 2023 cycle is the most recent NHANES release that simultaneously provides the full whole‐blood panel of lead, cadmium, mercury, selenium, and manganese together with fasting insulin and glucose, which are required to compute HOMA‐IR; it therefore offers the most current national picture of these exposures and of insulin resistance. Restricting to a single cycle also avoids pooling data across periods in which the laboratory methods, detection limits, and weighting frames for the metal assays differ, a source of artifactual heterogeneity when blood‐metal series are combined over time. This focus complements, rather than duplicates, the prior NHANES mediation analysis of Mao et al. [21], which used the older 2011 to 2016 cycles; analyzing the newest single cycle provides an updated and internally consistent estimate while sidestepping cross‐cycle assay drift. We acknowledge the corresponding trade‐off, namely, reduced sample size relative to a multi‐cycle pool, and we treat it among the study limitations.

The positive associations of selenium and manganese with HOMA‐IR warrant a cautious interpretation. Although the primary models showed a positive manganese association with apparent hs‐CRP mediation, this association did not survive adjustment for BMI (Section 3.6), so we do not regard it as a robust positive signal; any inflammatory contribution of manganese in these cross‐sectional data is at most modest and is confounded by adiposity. Mechanistically, manganese can activate the NLRP3 inflammasome at high concentrations [29], which would be consistent with a pro‐inflammatory effect, but our data do not provide robust support for such an effect on insulin resistance at population exposure levels. For selenium, supraphysiological concentrations have been associated with increased diabetes risk in observational studies and randomized trials [30], and the positive selenium estimate was directionally stable across adjustment, though selenium contributed negligible weight to the mixture. Across the five‐metal mixture, the overall effect remained inverse and was driven by lead, cadmium, and mercury both before and after adjustment.

The race/ethnicity‐stratified results showed consistent lead–IR associations across three major groups (non‐Hispanic White, non‐Hispanic Black, and Mexican American), suggesting that this relationship is not confounded by race‐specific factors. The nonsignificant finding in non‐Hispanic Asians likely reflects insufficient statistical power (*n* = 168) rather than a true null effect.

Because insulin resistance and cardiovascular disease (CVD) cluster together clinically and share metabolic determinants [31], we examined whether CVD could account for the metal–IR associations. Heavy metals are themselves recognized cardiovascular risk factors: Low‐level lead exposure predicts cardiovascular mortality in U.S. adults [32], cadmium is associated with peripheral arterial disease with sex differences in NHANES [33], and heavy metals together with inflammatory biomarkers are now framed among the novel risk factors for cardiovascular‐metabolic disease [34]. In our data, prevalent CVD was nearly twice as common among insulin‐resistant as among non‐resistant participants (12.9% vs. 7.0%), confirming the expected co‐occurrence. Adjusting for prevalent CVD nonetheless left every metal–IR estimate essentially unchanged (all changes ≤ 0.007 on the log scale; Section 3.6). We therefore interpret CVD as a downstream correlate of insulin resistance in this cross‐sectional sample rather than as a confounder of the metal–IR relationship; the reviewer's concern that unmeasured CVD might generate spurious associations is not supported by the data, although prospective designs would be needed to fully separate these intertwined endpoints.

This study has several strengths: The use of the most recent NHANES cycle, a formal mediation framework, sex‐stratified analysis, a WQS mixture assessment estimated with a standard optimizer, age‐ and race‐specific subgroup analyses, and a prespecified sensitivity series that directly probes adiposity and lifestyle confounding. Several limitations qualify the findings. First, the cross‐sectional design precludes causal inference and cannot establish the direction of the metal–IR associations. Second, the treatment of BMI is two‐sided: If adiposity lies on the reverse‐causation pathway, our primary models correctly avoid over‐adjustment, but if metals act through an adiposity‐independent inflammatory pathway, the primary models are subject to residual confounding by adiposity. The BMI‐adjusted sensitivity analyses bound this uncertainty but cannot resolve it. Third, blood metal concentrations reflect recent rather than lifelong exposure. Fourth, for the fasting biomarkers, we used the examination weight (WTMEC2YR) for consistency with the parent analysis rather than the fasting subsample weight (WTSAF2YR); a fully design‐correct fasting analysis would use WTSAF2YR, although the substantive findings are not expected to change. Fifth, the full lifestyle model (M4) was estimated on a reduced sample (*n* = 1,918) because day‐one dietary recall and family‐income data were missing for some participants, which limits its power and likely explains why mercury and the female lead estimate lost significance under M4 but not under M3. Sixth, the 2021 to 2023 physical‐activity questionnaire captures only recreational activity, so physical activity was adjusted using sedentary time and a recreational‐activity indicator rather than a complete metabolic‐equivalent score. Finally, we used a single inflammatory mediator (hs‐CRP); although hs‐CRP is the standard clinical‐laboratory marker of systemic inflammation, the inflammatory response involves multiple cytokines and pathways that a single biomarker cannot fully capture.

In conclusion, blood lead, cadmium, and mercury, individually and as a mixture, were inversely associated with insulin resistance in U.S. adults, and systemic inflammation measured by hs‐CRP accounted for a smaller but still statistically significant share of these associations after adjustment for adiposity. The inverse associations and the inflammatory mediation were substantially attenuated by BMI but not by smoking, a pattern consistent with adiposity‐driven reverse causation and hemodilution. The mediation was more pronounced in females and in younger adults. Given the cross‐sectional design, these findings are hypothesis‐generating: They are compatible with a contributory metal‐induced inflammatory pathway, but they cannot establish causal direction, and prospective studies with repeated biomarker measurement are needed to separate inflammatory mediation from adiposity‐driven confounding before anti‐inflammatory or exposure‐reduction strategies could be considered.

## Author Contributions

Cai Zhang: Conceptualization, Methodology, Formal analysis, Software, Visualization, Writing, original draft. Shidi Lin: Supervision, Writing, review and editing. Yu Lin: Data curation, Validation, Writing, review and editing.

## Conflicts of Interest

The authors declare no conflicts of interest.

## Supporting information


**Figure S1:** Fully adjusted associations of blood metals with insulin resistance, by sex.
**Figure S2:** WQS mixture weights for the metal‐mixture → HOMA‐IR association, by sex.
**Figure S3:** Proportion of each metal–HOMA‐IR association mediated by hs‐CRP, by sex.
**Figure S4:** Spearman correlation heatmap of metals, hs‐CRP, and glycemic biomarkers.
**Figure S5:** Age‐stratified hs‐CRP mediation of the lead → HOMA‐IR association.
**Figure S6:** Attenuation of metal–log(HOMA‐IR) associations across nested adjustment models.
**Table S1:** Participant characteristics stratified by sex, NHANES 2021–2023.
**Table S2:** Metal–log(HOMA‐IR) β estimates across progressive adjustment models.
**Table S3:** Spearman correlation matrix of metals, hs‐CRP, and glycemic biomarkers.
**Table S4:** Age‐stratified mediation of the lead → HOMA‐IR association by hs‐CRP.
**Table S5:** Sensitivity analysis of metal–log(HOMA‐IR) associations under progressive adjustment.
**Table S6:** Decomposition of the hs‐CRP indirect effect by metal and group.
**Table S7:** Weighted quantile sum (WQS) mixture associations with log(HOMA‐IR) under progressive adjustment.
**Table S8:** hs‐CRP mediation of metal–HOMA‐IR associations before and after BMI and smoking adjustment.
**Table S9:** Prevalent cardiovascular disease by insulin‐resistance status, with and without CVD adjustment.

## Data Availability

Data used in this study are publicly available from the National Health and Nutrition Examination Survey website (https://wwwn.cdc.gov/nchs/nhanes/). Analysis code is available from the corresponding author upon reasonable request.

## References

[1] A. Menke , S. Casagrande , L. Geiss , and C. C. Cowie , “Prevalence of and Trends in Diabetes Among Adults in the United States, 1988–2012,” JAMA 314 (2015): 1021–1029, 10.1001/jama.2015.10029. 26348752

[2] H. Kolb and S. Martin , “Environmental/Lifestyle Factors in the Pathogenesis and Prevention of Type 2 Diabetes,” BMC Medicine 15 (2017): 131, 10.1186/s12916-017-0901-x. 28720102 PMC5516328

[3] S. S. Moon , “Association of Lead, Mercury and Cadmium With Diabetes in the Korean Population,” Diabetic Medicine 30 (2013): e143–e148, 10.1111/dme.12103. 23278294

[4] A. A. Tinkov , T. Filippini , O. P. Ajsuvakova , et al., “The Role of Cadmium in Obesity and Diabetes,” Science of the Total Environment 601–602 (2017): 741–755, 10.1016/j.scitotenv.2017.05.224. 28577409

[5] A. Menke , E. Guallar , and C. C. Cowie , “Metals in Urine and Diabetes in U.S. Adults,” Diabetes 65 (2016): 164–171, 10.2337/db15-0316. 26542316 PMC4686948

[6] Q. Wu , R. Gan , S. Chen , et al., “Associations of Metal Mixtures With Insulin Resistance and Glucose Homeostasis in U.S. Adults From the NHANES 2011–2018,” Scientific Reports 16 (2026): 2047, 10.1038/s41598-025-31637-3. 41540139 PMC12808658

[7] K. Jomova and M. Valko , “Advances in Metal‐Induced Oxidative Stress and Human Disease,” Toxicology 283 (2011): 65–87, 10.1016/j.tox.2011.03.001. 21414382

[8] E. Karpuzoglu , S. D. Holladay , and R. M. Gogal , “NLRP3 Inflammasome Dysregulation by Endocrine‐Disrupting Chemicals and Heavy Metals: Developmental Programming, Sex Differences, and Inflammaging Across the Lifespan,” International Immunopharmacology 182 (2026): 116792, 10.1016/j.intimp.2026.116792. 42105707

[9] M. Y. Donath and S. E. Shoelson , “Type 2 Diabetes as an Inflammatory Disease,” Nature Reviews. Immunology 11 (2011): 98–107, 10.1038/nri2925. 21233852

[10] G. S. Hotamisligil , “Inflammation and Metabolic Disorders,” Nature 444 (2006): 860–867, 10.1038/nature05485. 17167474

[11] G. S. Hotamisligil , P. Peraldi , A. Budavari , R. Ellis , M. F. White , and B. M. Spiegelman , “IRS‐1‐Mediated Inhibition of Insulin Receptor Tyrosine Kinase Activity in TNF‐Alpha‐ and Obesity‐Induced Insulin Resistance,” Science 271 (1996): 665–668, 10.1126/science.271.5249.665. 8571133

[12] A. R. Saltiel and J. M. Olefsky , “Inflammatory Mechanisms Linking Obesity and Metabolic Disease,” Journal of Clinical Investigation 127 (2017): 1–4, 10.1172/JCI92035. 28045402 PMC5199709

[13] A. B. Goldfine , V. Fonseca , and S. E. Shoelson , “Therapeutic Approaches to Target Inflammation in Type 2 Diabetes,” Clinical Chemistry 57 (2011): 162–167, 10.1373/clinchem.2010.148833. 21098138 PMC3227024

[14] A. D. Pradhan , J. E. Manson , N. Rifai , J. E. Buring , and P. M. Ridker , “C‐Reactive Protein, Interleukin 6, and Risk of Developing Type 2 Diabetes Mellitus,” JAMA 286 (2001): 327–334, 10.1001/jama.286.3.327. 11466099

[15] G. R. Romeo , J. Lee , and S. E. Shoelson , “Metabolic Syndrome, Insulin Resistance, and Roles of Inflammation: Mechanisms and Therapeutic Targets,” Arteriosclerosis, Thrombosis, and Vascular Biology 32 (2012): 1771–1776, 10.1161/ATVBAHA.111.241869. 22815343 PMC4784686

[16] S. U. Baek and J. H. Yoon , “Association Between Heavy Metals Exposure and Elevated High‐Sensitivity C‐Reactive Protein: Mediating Role of Body Mass Index,” Biomolecules 15 (2025): 1491, 10.3390/biom15111491. 41301410 PMC12650004

[17] E. Obeng‐Gyasi and B. Obeng‐Gyasi , “Association of Combined Lead, Cadmium, and Mercury With Systemic Inflammation,” Frontiers in Public Health 12 (2024): 1385500, 10.3389/fpubh.2024.1385500. 39267632 PMC11390544

[18] P. J. Little , S. Offermanns , H. Cho , et al., “Insulin Resistance: An Update on Biochemical and Pathophysiological Mechanisms and Impact on Various Diseases,” iNew Medicine 2 (2026): e70028, 10.1002/inm3.70028.

[19] K. J. Preacher and A. F. Hayes , “Asymptotic and Resampling Strategies for Assessing and Comparing Indirect Effects in Multiple Mediator Models,” Behavior Research Methods 40 (2008): 879–891, 10.3758/BRM.40.3.879. 18697684

[20] C. Carrico , C. Gennings , D. C. Wheeler , and P. Factor‐Litvak , “Characterization of Weighted Quantile Sum Regression for Highly Correlated Data in a Risk Analysis Setting,” Journal of Agricultural, Biological, and Environmental Statistics 20 (2015): 100–120, 10.1007/s13253-014-0180-3. 30505142 PMC6261506

[21] Q. Mao , X. Zhang , X. Zhu , X. Tian , and Y. Kong , “Inflammation Factors Mediate the Association Between Heavy Metal and HOMA‐IR Index: An Integrated Approach From the NHANES (2011∼2016),” American Journal of the Medical Sciences 370 (2025): 19–29, 10.1016/j.amjms.2025.03.013. 40158727

[22] CDC , “National Health and Nutrition Examination Survey: 2021–2023 Laboratory Data Documentation,” 2024 accessed 15 March 2026, https://wwwn.cdc.gov/nchs/nhanes/.

[23] D. R. Matthews , J. P. Hosker , A. S. Rudenski , B. A. Naylor , D. F. Treacher , and R. C. Turner , “Homeostasis Model Assessment: Insulin Resistance and Beta‐Cell Function From Fasting Plasma Glucose and Insulin Concentrations in Man,” Diabetologia 28 (1985): 412–419, 10.1007/BF00280883. 3899825

[24] P. M. Ridker , “Clinical Application of C‐Reactive Protein for Cardiovascular Disease Detection and Prevention,” Circulation 107 (2003): 363–369, 10.1161/01.CIR.0000053730.47739.3C. 12551853

[25] M. A. Padilla , M. Elobeid , D. M. Ruden , and D. B. Allison , “An Examination of the Association of Selected Toxic Metals With Total and Central Obesity Indices,” International Journal of Environmental Research and Public Health 7 (2010): 3332–3347, 10.3390/ijerph7093332. 20948927 PMC2954548

[26] T. Shen , L. Zhong , G. Ji , et al., “Associations Between Metal(Loid) Exposure With Overweight and Obesity and Abdominal Obesity in the General Population: A Cross‐Sectional Study in China,” Chemosphere 350 (2024): 140963, 10.1016/j.chemosphere.2023.140963. 38114022

[27] R. L. Grubb , A. Black , G. Izmirlian , et al., “Serum Prostate‐Specific Antigen Hemodilution Among Obese Men Undergoing Screening in the Prostate, Lung, Colorectal, and Ovarian Cancer Screening Trial,” Cancer Epidemiology, Biomarkers & Prevention 18 (2009): 748–751, 10.1158/1055-9965.EPI-08-0938. 19258472

[28] J. Zhao , J. Y. Y. Leung , S. L. Lin , and C. M. Schooling , “Cigarette Smoking and Testosterone in Men and Women: A Systematic Review and Meta‐Analysis of Observational Studies,” Preventive Medicine 85 (2016): 1–10, 10.1016/j.ypmed.2015.12.021. 26763163

[29] S. Sarkar , D. Rokad , E. Malovic , et al., “Manganese Activates NLRP3 Inflammasome Signaling and Propagates Exosomal Release of ASC in Microglial Cells,” Science Signaling 12 (2019): eaat9900, 10.1126/scisignal.aat9900. 30622196 PMC6420319

[30] S. Stranges , J. R. Marshall , R. Natarajan , et al., “Effects of Long‐Term Selenium Supplementation on the Incidence of Type 2 Diabetes,” Annals of Internal Medicine 147 (2007): 217–223, 10.7326/0003-4819-147-4-200708210-00175. 17620655

[31] R. A. DeFronzo and E. Ferrannini , “Insulin Resistance: A Multifaceted Syndrome Responsible for NIDDM, Obesity, Hypertension, Dyslipidemia, and Atherosclerotic Cardiovascular Disease,” Diabetes Care 14 (1991): 173–194, 10.2337/diacare.14.3.173. 2044434

[32] B. P. Lanphear , S. Rauch , P. Auinger , R. W. Allen , and R. W. Hornung , “Low‐Level Lead Exposure and Mortality in US Adults: A Population‐Based Cohort Study,” Lancet Public Health 3 (2018): e177–e184, 10.1016/S2468-2667(18)30025-2. 29544878

[33] M. Tellez‐Plaza , A. Navas‐Acien , C. M. Crainiceanu , A. R. Sharrett , and E. Guallar , “Cadmium and Peripheral Arterial Disease: Gender Differences in the 1999–2004 US National Health and Nutrition Examination Survey,” American Journal of Epidemiology 172 (2010): 671–681, 10.1093/aje/kwq172. 20693268 PMC2950816

[34] Z. Wang , S. Chen , S. Xu , and J. Weng , “Risk Factors for Cardiovascular and Metabolic Disease: Integrating Traditional and Novel Paradigms,” iNew Medicine 1 (2025): e70000, 10.1002/inm3.70000.

