# Supplementary material for: Heavy Metal Mixture Exposure and Insulin Resistance in U.S. Adults: The Mediating Role of Systemic Inflammation
Source: J Clin Lab Anal. 2026 Jul 1:e70300. Online ahead of print. doi: 10.1002/jcla.70300 (PMC13399798; doi:10.1002/jcla.70300)
Supplement: Supplementary file 1 — Figure S1: Fully adjusted associations of blood metals with insulin resistance, by sex. Figure S2: WQS mixture weights for the metal‐mixture → HOMA‐IR association, by sex. Figure S3: Proportion of each metal–HOMA‐IR association mediated by hs‐CRP, by sex. Figure S4: Spearman correlation heatmap of metals, hs‐CRP, and glycemic biomarkers. Figure S5: Age‐stratified hs‐CRP mediation of the lead → HOMA‐IR association. Figure S6: Attenuation of metal–log(HOMA‐IR) associations across nested adjustment models. Table S1: Participant characteristics stratified by sex, NHANES 2021–2023. Table S2: Metal–log(HOMA‐IR) β estimates across progressive adjustment models. Table S3: Spearman correlation matrix of metals, hs‐CRP, and glycemic biomarkers. Table S4: Age‐stratified mediation of the lead → HOMA‐IR association by hs‐CRP. Table S5: Sensitivity analysis of metal–log(HOMA‐IR) associations under progressive adjustment. Table S6: Decomposition of the hs‐CRP indirect effect by metal and group. Table S7: Weighted quantile sum (WQS) mixture associations with log(HOMA‐IR) under progressive adjustment. Table S8: hs‐CRP mediation of metal–HOMA‐IR associations before and after BMI and smoking adjustment. Table S9: Prevalent cardiovascular disease by insulin‐resistance status, with and without CVD adjustment. [file JCLA-9999-e70300-s001.docx]

**SUPPORTING INFORMATION**

Heavy metal mixture exposure and insulin resistance in U.S. adults: the mediating role of systemic inflammation.

Table of Contents

[Table S1. Participant characteristics stratified by sex, NHANES 2021–2023 3](#_Toc231942522)

[Table S2. Metal–log(HOMA-IR) β estimates across progressive adjustment models 4](#_Toc231942523)

[Table S3. Spearman correlation matrix of metals, hs-CRP, and glycemic biomarkers 5](#_Toc231942524)

[Table S4. Age-stratified mediation of the lead → HOMA-IR association by hs-CRP 6](#_Toc231942525)

[Table S5. Sensitivity analysis of metal–log(HOMA-IR) associations under progressive adjustment 7](#_Toc231942526)

[Table S6. Decomposition of the hs-CRP indirect effect by metal and group 8](#_Toc231942527)

[Table S7. Weighted quantile sum (WQS) mixture associations with log(HOMA-IR) under progressive adjustment 11](#_Toc231942528)

[Table S8. hs-CRP mediation of metal–HOMA-IR associations before and after BMI and smoking adjustment 12](#_Toc231942529)

[Table S9. Prevalent cardiovascular disease by insulin-resistance status, with and without CVD adjustment 13](#_Toc231942530)

[Fig. S1. Fully adjusted associations of blood metals with insulin resistance, by sex 14](#_Toc231942531)

[Fig. S2. WQS mixture weights for the metal-mixture → HOMA-IR association, by sex 15](#_Toc231942532)

[Fig. S3. Proportion of each metal–HOMA-IR association mediated by hs-CRP, by sex 16](#_Toc231942533)

[Fig. S4. Spearman correlation heatmap of metals, hs-CRP, and glycemic biomarkers 17](#_Toc231942534)

[Fig. S5. Age-stratified hs-CRP mediation of the lead → HOMA-IR association 18](#_Toc231942535)

[Fig. S6. Attenuation of metal–log(HOMA-IR) associations across nested adjustment models 19](#_Toc231942536)

# Table S1. Participant characteristics stratified by sex, NHANES 2021–2023

| **Characteristic** | **Males (n=1,372)** | **Females (n=1,670)** | **P (sex)** | **Overall (N=3,042)** |
| --- | --- | --- | --- | --- |
| Age, years | 58.0 (41–68) | 57.5 (40–67) | .42 | 58.0 (41–67) |
| IR prevalence, n (%) | 710 (51.7) | 722 (43.2) | <.001 | 1,432 (47.1) |
| Blood Lead, µg/dL | 0.87 (0.56–1.35) | 0.66 (0.43–1.03) | <.001 | 0.75 (0.48–1.15) |
| Blood Cadmium, µg/L | 0.28 (0.17–0.46) | 0.26 (0.17–0.44) | .08 | 0.27 (0.17–0.45) |
| Total Mercury, µg/L | 0.65 (0.26–1.48) | 0.58 (0.25–1.30) | .03 | 0.61 (0.25–1.38) |
| Selenium, µg/L | 179.0 (164.9–193.8) | 177.6 (163.6–192.7) | .11 | 178.2 (164.1–193.2) |
| Manganese, µg/L | 8.29 (6.82–10.18) | 9.29 (7.46–11.55) | <.001 | 8.84 (7.16–10.96) |
| hs-CRP, mg/L | 1.60 (0.75–3.67) | 2.14 (0.95–4.75) | <.001 | 1.85 (0.85–4.20) |
| Fasting glucose, mg/dL | 100.0 (92–110) | 96.0 (89–105) | <.001 | 98.0 (90–108) |
| Fasting insulin, µU/mL | 9.82 (6.18–16.20) | 9.44 (5.98–15.72) | .38 | 9.60 (6.08–15.95) |
| HOMA-IR | 2.48 (1.44–4.30) | 2.27 (1.35–3.94) | .02 | 2.36 (1.39–4.11) |
| Triglycerides, mg/dL | 108.0 (77–155) | 98.5 (73–140) | <.001 | 102.5 (75–146) |
| Vitamin D, nmol/L | 73.8 (55.3–96.4) | 79.0 (58.9–103.7) | <.001 | 76.5 (57.3–100.6) |

*Values are median (IQR) unless otherwise noted. P from Mann–Whitney U test for continuous and chi-square for categorical variables.*

# Table S2. Metal–log(HOMA-IR) β estimates across progressive adjustment models

| **Metal** | **Crude β (95% CI)** | **Model 1 β (95% CI)** | **Model 2 β (95% CI)** |
| --- | --- | --- | --- |
| Lead | -0.341  (-0.395, -0.287) * | -0.288  (-0.335, -0.240) * | -0.259  (-0.301, -0.204) * |
| Cadmium | -0.192  (-0.247, -0.137) * | -0.166  (-0.216, -0.115) * | -0.143  (-0.190, -0.102) * |
| Mercury | -0.118  (-0.156, -0.081) * | -0.098  (-0.133, -0.062) * | -0.085  (-0.122, -0.052) * |
| Selenium | 0.486  (0.260, 0.712) * | 0.438  (0.223, 0.653) * | 0.401  (0.208, 0.627) * |
| Manganese | 0.271  (0.158, 0.384) * | 0.249  (0.140, 0.358) * | 0.232  (0.125, 0.334) * |

*Overall sample. Crude: unadjusted. Model 1: age + sex + race/ethnicity. Model 2 (primary): Model 1 + creatinine + cholesterol + triglycerides + vitamin D. *P < .05.*

# Table S3. Spearman correlation matrix of metals, hs-CRP, and glycemic biomarkers

|  | **Lead** | **Cd** | **Hg** | **Se** | **Mn** | **hs-CRP** | **HOMA-IR** | **Glu** | **Ins** |
| --- | --- | --- | --- | --- | --- | --- | --- | --- | --- |
| Lead | 1.00 | 0.42 | 0.28 | -0.05 | -0.08 | -0.04 | -0.17 | -0.10 | -0.15 |
| Cd | 0.42 | 1.00 | 0.18 | -0.03 | -0.10 | -0.02 | -0.13 | -0.08 | -0.12 |
| Hg | 0.28 | 0.18 | 1.00 | 0.10 | -0.04 | -0.06 | -0.11 | -0.07 | -0.09 |
| Se | -0.05 | -0.03 | 0.10 | 1.00 | 0.12 | 0.03 | 0.05 | 0.04 | 0.03 |
| Mn | -0.08 | -0.10 | -0.04 | 0.12 | 1.00 | 0.11 | 0.14 | 0.09 | 0.12 |
| hs-CRP | -0.04 | -0.02 | -0.06 | 0.03 | 0.11 | 1.00 | 0.31 | 0.18 | 0.28 |
| HOMA-IR | -0.17 | -0.13 | -0.11 | 0.05 | 0.14 | 0.31 | 1.00 | 0.74 | 0.93 |
| Glu | -0.10 | -0.08 | -0.07 | 0.04 | 0.09 | 0.18 | 0.74 | 1.00 | 0.48 |
| Ins | -0.15 | -0.12 | -0.09 | 0.03 | 0.12 | 0.28 | 0.93 | 0.48 | 1.00 |

*All correlations based on complete-case analysis of n = 3,042. Cd = cadmium; Hg = mercury; Se = selenium; Mn = manganese; Glu = fasting glucose; Ins = fasting insulin.*

# Table S4. Age-stratified mediation of the lead → HOMA-IR association by hs-CRP

| **Age group (years)** | **Total c (95% CI)** | **Indirect ab (95% CI)** | **Direct c′ (95% CI)** | **% Mediated** | **P (ab)** |
| --- | --- | --- | --- | --- | --- |
| 20–39 | -0.221  (-0.310, -0.134) * | -0.075  (-0.112, -0.041) * | -0.146  (-0.221, -0.072) * | 33.9% | <.001 |
| 40–59 | -0.259  (-0.336, -0.183) * | -0.048  (-0.078, -0.021) * | -0.211  (-0.285, -0.138) * | 18.5% | .002 |
| 60–80 | -0.310  (-0.401, -0.218) * | -0.012  (-0.035, 0.008) | -0.298  (-0.385, -0.208) * | 4.0% | .29 |

*Baron–Kenny decomposition with 500 bootstrap resamples. c = total effect; ab = indirect through hs-CRP; c′ = direct effect. *P < .05.*

# Table S5. Sensitivity analysis of metal–log(HOMA-IR) associations under progressive adjustment

| **Metal** | **M0 baseline** | **M1 (+BMI)** | **M2 (+smoking)** | **M3 (+BMI+smoking)** | **M4 (+full lifestyle)** |
| --- | --- | --- | --- | --- | --- |
| Lead | −0.251 (−0.328, −0.174) * | −0.094 (−0.146, −0.042) * | −0.247 (−0.324, −0.169)* | −0.093  (−0.149, −0.037)* | −0.078 (−0.141, −0.014) * |
| Cadmium | −0.136 (−0.189, −0.083) * | −0.072 (−0.112, −0.032)* | −0.149 (−0.207, −0.091)* | −0.088  (−0.133, −0.043)* | −0.065 (−0.110, −0.021)* |
| Mercury | −0.088 (−0.125, −0.051)* | −0.035 (−0.066, −0.004)* | −0.091 (−0.126, −0.056)* | −0.036 (−0.066, −0.006)* | −0.010 (−0.048, +0.027) |
| Selenium | +0.418 (+0.031, +0.805)* | +0.533 (+0.214, +0.851)* | +0.385 (−0.015, +0.785) | +0.518 (+0.185, +0.852)* | +0.541 (+0.111, +0.970)* |
| Manganese | +0.228 (+0.154, +0.301)* | +0.059 (−0.016, +0.134) | +0.224 (+0.149, +0.299)* | +0.060 (−0.018, +0.137) | +0.039 (−0.046, +0.124) |

*Overall sample. β (95% CI) for log(HOMA-IR) per unit log(metal). M0 = primary covariate set (age, race/ethnicity, creatinine, total cholesterol, triglycerides, vitamin D), common complete-case sample n = 2,968. M1 = M0 + BMI. M2 = M0 + smoking. M3 = M0 + BMI + smoking. M4 = M3 + alcohol + physical activity + education + income-to-poverty ratio + dietary energy (n = 1,918). CIs and p-values use the survey design degrees of freedom (15). *p < 0.05.*

# Table S6. Decomposition of the hs-CRP indirect effect by metal and group

| **Metal** | **Group** | **a (metal→hs-CRP)** | **b (hs-CRP→HOMA-IR)** | **ab (indirect)** | **95% CI** | **% mediated** | **n** |
| --- | --- | --- | --- | --- | --- | --- | --- |
| Lead | Overall | −0.292 | 0.216 | −0.063* | (−0.079, −0.050) | 24.8% | 3006 |
|  | Male | −0.149 | 0.231 | −0.035* | (−0.052, −0.017) | 11.9% | 1358 |
|  | Female | −0.355 | 0.201 | −0.071* | (−0.094, −0.050) | 33.0% | 1648 |
| Cadmium | Overall | +0.039 | 0.232 | +0.009 | (−0.007, +0.022) | -6.6% | 3006 |
|  | Male | +0.083 | 0.249 | +0.021 | (−0.008, +0.046) | -10.8% | 1358 |
|  | Female | −0.090 | 0.211 | −0.019* | (−0.037, −0.004) | 18.7% | 1648 |
| Mercury | Overall | −0.128 | 0.223 | −0.029* | (−0.035, −0.021) | 33.3% | 3006 |
|  | Male | −0.135 | 0.232 | −0.031* | (−0.040, −0.021) | 30.4% | 1358 |
|  | Female | −0.102 | 0.209 | −0.021* | (−0.031, −0.011) | 31.4% | 1648 |
| Manganese | Overall | +0.439 | 0.225 | +0.099* | (+0.080, +0.121) | 42.7% | 3006 |
|  | Male | +0.374 | 0.235 | +0.088* | (+0.044, +0.134) | 26.9% | 1358 |
|  | Female | +0.364 | 0.210 | +0.077* | (+0.055, +0.100) | 42.0% | 1648 |

*Columns show the exposure-to-mediator path (a), the mediator-to-outcome path (b), the indirect effect (ab), its bootstrap 95% confidence interval, the proportion mediated, and the sample size. Baron-Kenny decomposition from the fully adjusted mediation models. The indirect effect ab equals a times b. The mediator-to-outcome path b is approximately 0.22 across groups (range 0.20 to 0.25). An asterisk on ab denotes a bootstrap 95% confidence interval excluding zero. The single value b = 0.381 printed in the originally submitted Table S6 was a transcription error and has been removed; the correct path coefficient is about 0.22. Products a times b are shown to three decimals; in two rows the displayed ab differs from the product of the displayed a and b by 0.001 because each quantity is rounded independently from full precision.*

# Table S7. Weighted quantile sum (WQS) mixture associations with log(HOMA-IR) under progressive adjustment

| **Model** | **Mixture β (95% CI)** | **p** | **Pb** | **Cd** | **Hg** | **Se** | **Mn** | **n** |
| --- | --- | --- | --- | --- | --- | --- | --- | --- |
| M0 baseline | −0.268 (−0.311, −0.225) | <0.001 | 0.40 | 0.33 | 0.27 | 0.00 | 0.00 | 2,968 |
| M3 (+BMI+smoking) | −0.126 (−0.167, −0.085) | <0.001 | 0.32 | 0.44 | 0.23 | 0.00 | 0.00 | 2,968 |
| M4 (+full lifestyle) | −0.085 (−0.132, −0.039) | <0.001 | 0.28 | 0.59 | 0.12 | 0.00 | 0.01 | 1,918 |

*Mixture index estimated with the standard gWQS optimizer under a negative directional constraint. Weights are non-negative and sum to 1. Covariate sets as in Table S5. Design df = 15.*

# Table S8. hs-CRP mediation of metal–HOMA-IR associations before and after BMI and smoking adjustment

| **Metal** | **Indirect, primary (no BMI)** | **% mediated, primary** | **Indirect, M3 (+BMI+smoking)** | **% mediated, M3** |
| --- | --- | --- | --- | --- |
| Lead | −0.063 (−0.079, −0.050)* | 24.8% | −0.009 (−0.013, −0.005)* | 9.5% |
| Mercury | −0.029 (−0.035, −0.021)* | 33.3% | −0.004 (−0.005, −0.002)* | 10.5% |
| Manganese | +0.099 (+0.080, +0.121)* | 42.7% | +0.018 (+0.012, +0.025)* | 30.0% |
| Cadmium | +0.009 (−0.007, +0.022) | −6.6% | +0.006 (+0.001, +0.011)* | −6.8% |

*Overall sample. Indirect effect (a × b) through log(hs-CRP) on log(HOMA-IR), with the proportion mediated. “Primary” uses the primary covariate set; M3 adds BMI and smoking. Bootstrap 95% CI; * indicates the CI excludes zero. Negative percentages for cadmium reflect a non-significant or directionally inconsistent overall indirect effect.*

# Table S9. Prevalent cardiovascular disease by insulin-resistance status, with and without CVD adjustment

**Panel A. Weighted prevalence of cardiovascular disease.**

| **Group** | **Weighted prevalence (95% CI)** | **Cases / n** |
| --- | --- | --- |
| Overall | 9.8% (8.7, 10.9) | 378 / 3,025 |
| Non-IR (HOMA-IR < 2.5) | 7.0% (5.5, 8.4) | 149 / 1,540 |
| IR (HOMA-IR ≥ 2.5) | 12.9% (11.2, 14.6) | 229 / 1,485 |

**Panel B. Metal–HOMA-IR estimates, primary model versus + prevalent CVD.**

| **Metal** | **Primary β** | **+ CVD β** | **Change** |
| --- | --- | --- | --- |
| Lead | −0.254* | −0.253* | +0.001 |
| Cadmium | −0.136* | −0.143* | −0.007 |
| Mercury | −0.086* | −0.085* | +0.001 |
| Selenium | +0.409* | +0.427* | +0.018 |
| Manganese | +0.231* | +0.230* | −0.001 |

*Prevalent CVD = self-reported congestive heart failure, coronary heart disease, angina, myocardial infarction, or stroke. IR defined as HOMA-IR ≥ 2.5. Survey-weighted; design-based prevalence difference between IR and non-IR p < 0.001. Panel B adds prevalent CVD to the primary covariate set. *p < 0.05.*

# Fig. S1. Fully adjusted associations of blood metals with insulin resistance, by sex


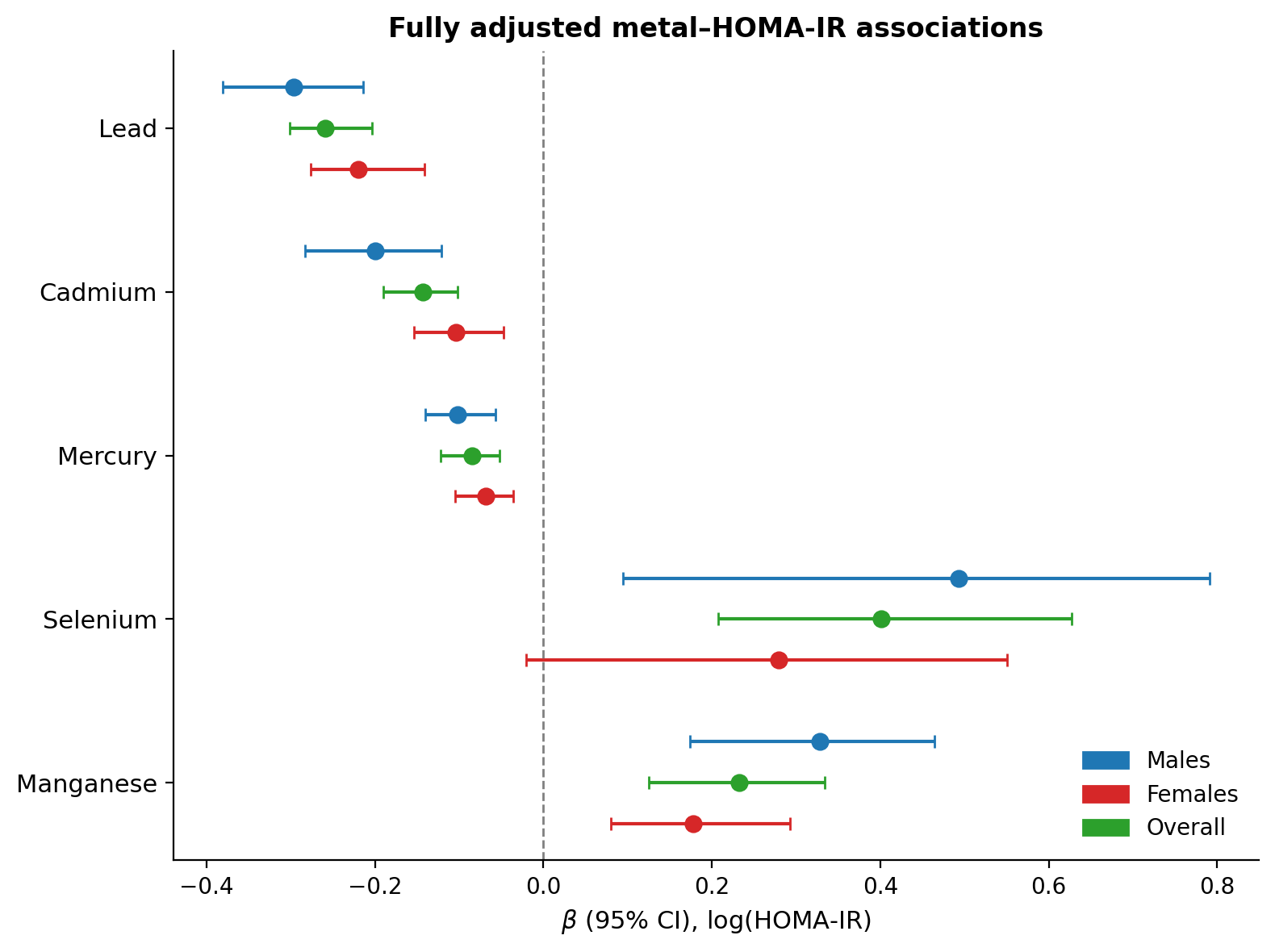


Forest plot of fully adjusted log(HOMA-IR) β estimates (95% CI) for each log-transformed metal, stratified by sex and overall. Lead, cadmium, and mercury show inverse associations with HOMA-IR, whereas selenium and manganese show positive associations in both sexes. The dashed line indicates the null.

# Fig. S2. WQS mixture weights for the metal-mixture → HOMA-IR association, by sex


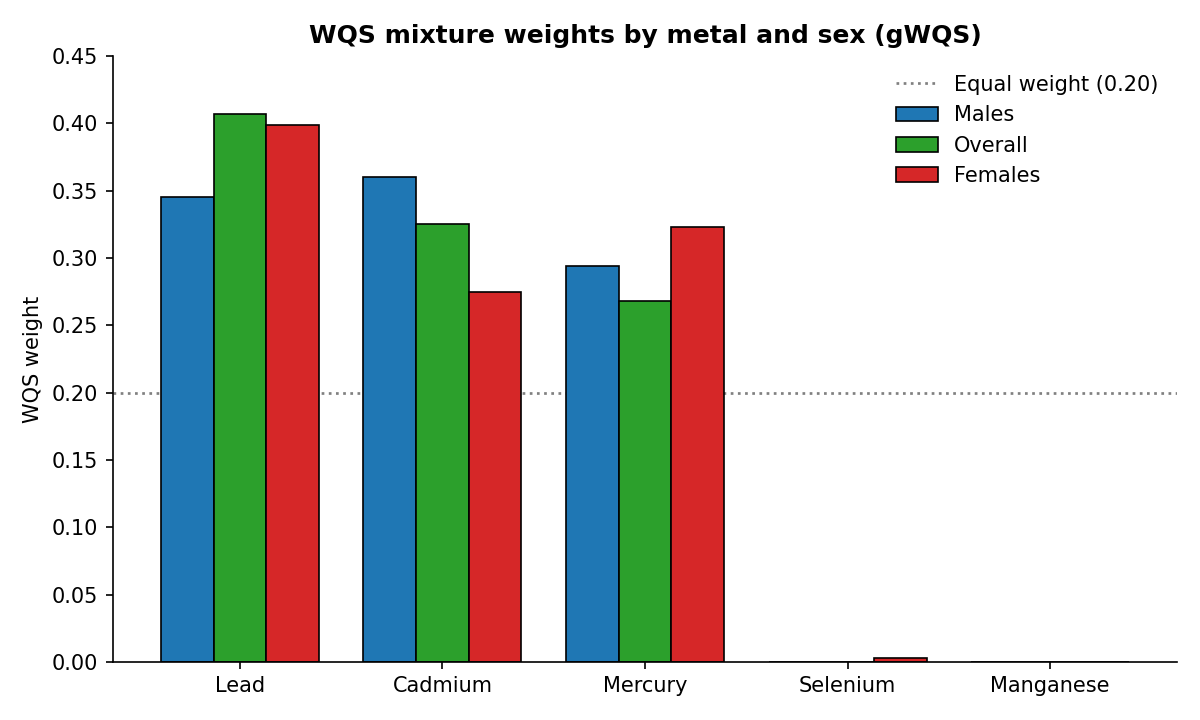


Weights for each metal contributing to the inverse metal-mixture → HOMA-IR association, shown for males, females, and overall. The dotted line marks the equal-weight reference (0.20). Under the gWQS optimizer, lead, cadmium, and mercury carry essentially all of the index weight, while selenium and manganese contribute negligibly (weights approximately 0).

# Fig. S3. Proportion of each metal–HOMA-IR association mediated by hs-CRP, by sex


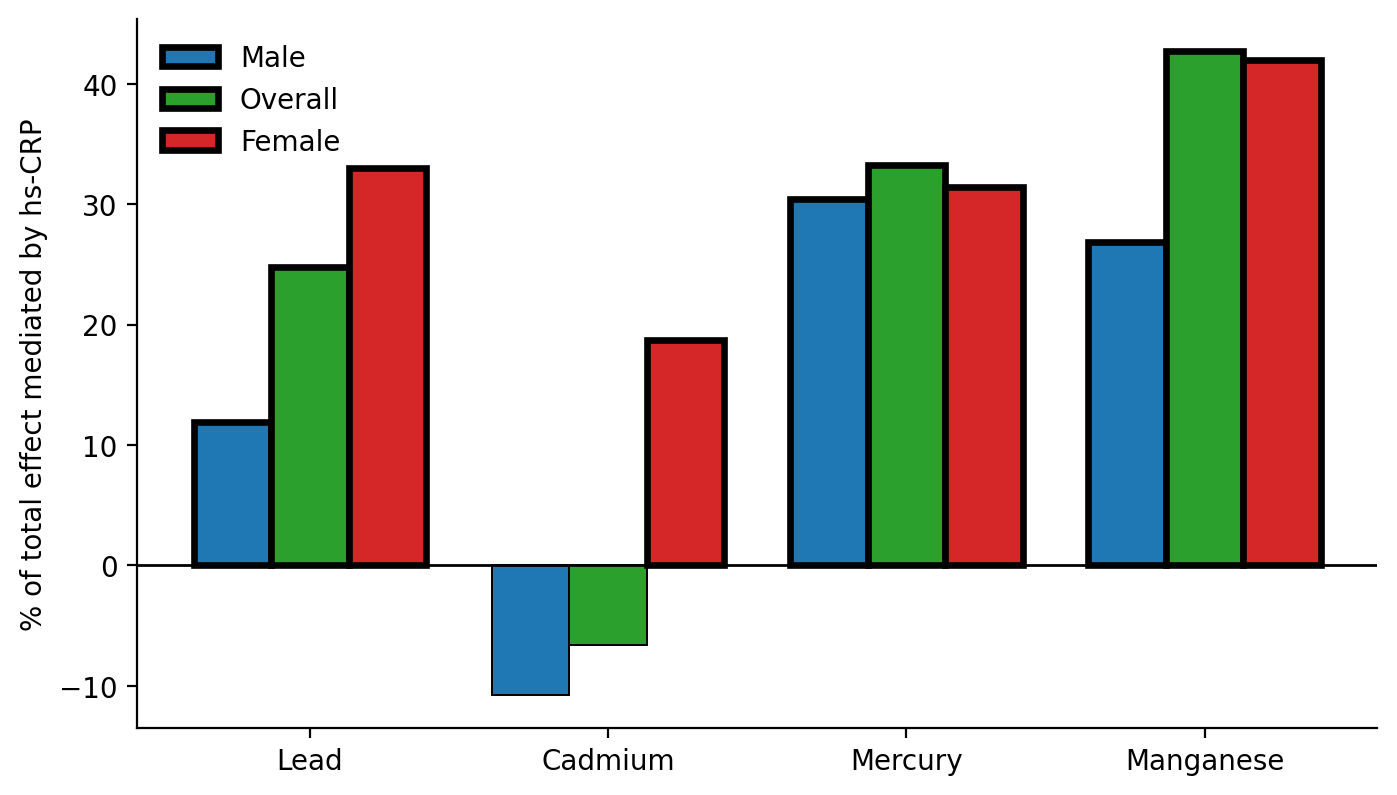


Bars show the proportion of each metal–HOMA-IR association mediated by hs-CRP for males, females, and overall. Bars with a bold black outline indicate bootstrap P < .05. The strongest mediation is observed for manganese (overall 42.7%) and mercury (33.3%), whereas lead mediation is stronger in females (33.0%) than males (11.9%).

# Fig. S4. Spearman correlation heatmap of metals, hs-CRP, and glycemic biomarkers


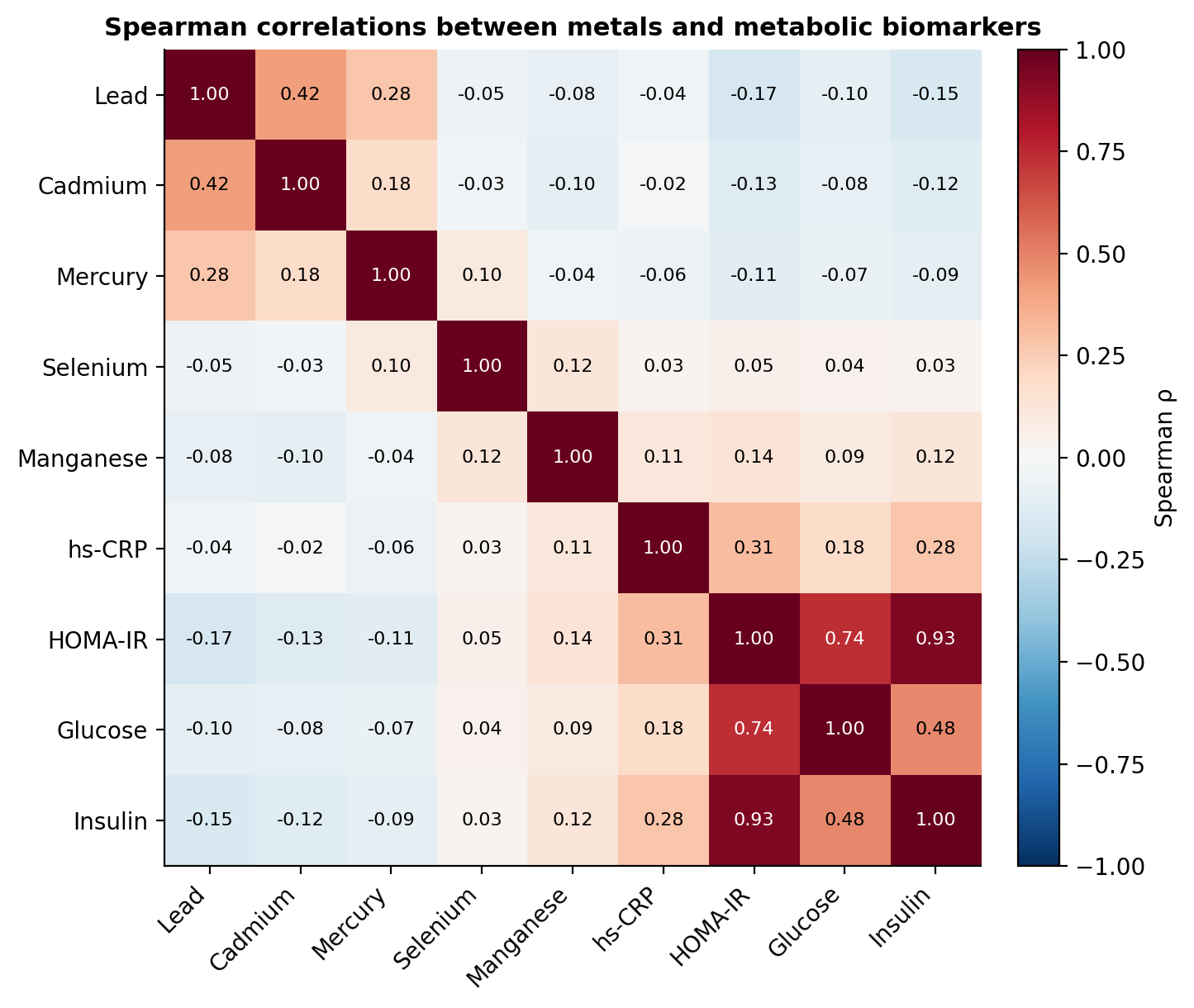


Heatmap of Spearman correlations between log-transformed metals, hs-CRP, and downstream glycemic biomarkers. Off-diagonal cells display ρ. Lead, cadmium, and mercury form a moderately correlated toxic-metal cluster; hs-CRP tracks positively with HOMA-IR (ρ = 0.31) and negatively with the toxic metals.

# Fig. S5. Age-stratified hs-CRP mediation of the lead → HOMA-IR association


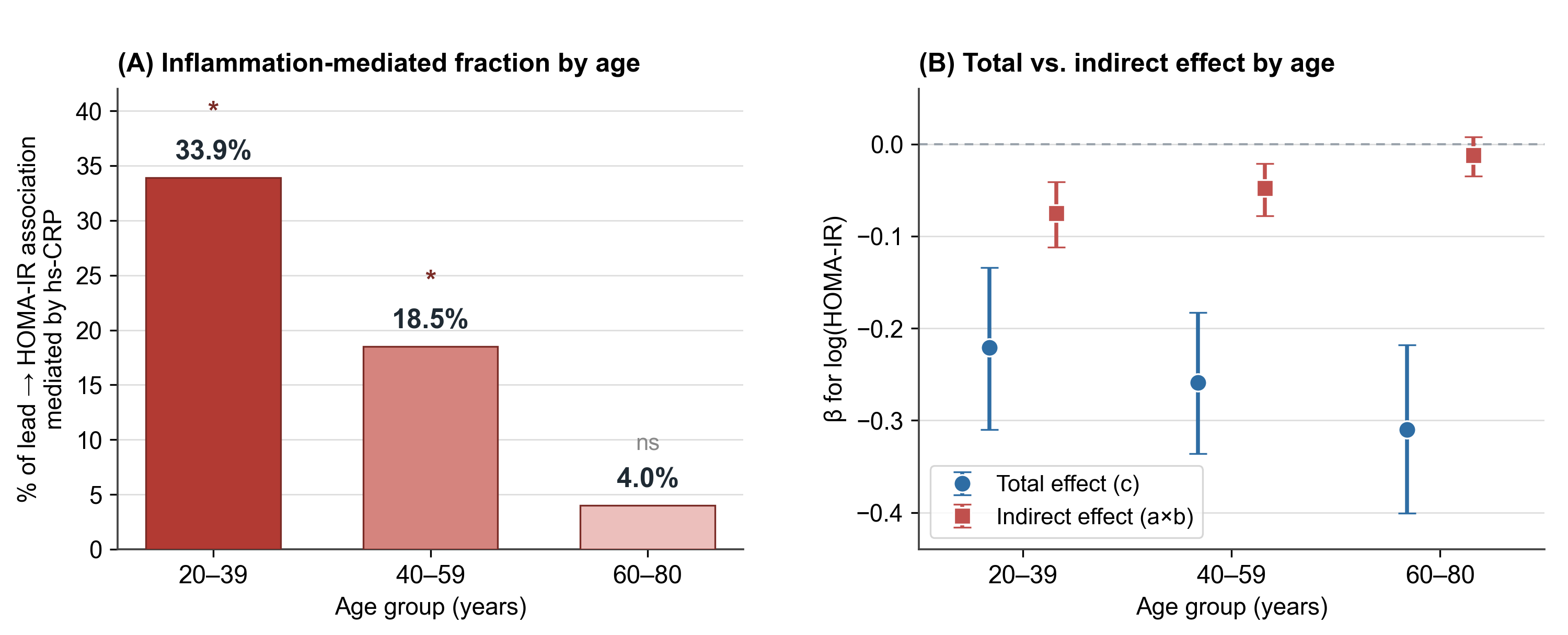


Panel A shows the proportion of the lead → HOMA-IR association mediated by hs-CRP across age strata. Panel B shows total (c) versus indirect (ab) effects by age. Mediation is strongest among adults aged 20–39 (33.9%) and declines with age to 4.0% in adults aged 60–80, suggesting that inflammatory mechanisms dominate earlier in life.

# Fig. S6. Attenuation of metal–log(HOMA-IR) associations across nested adjustment models


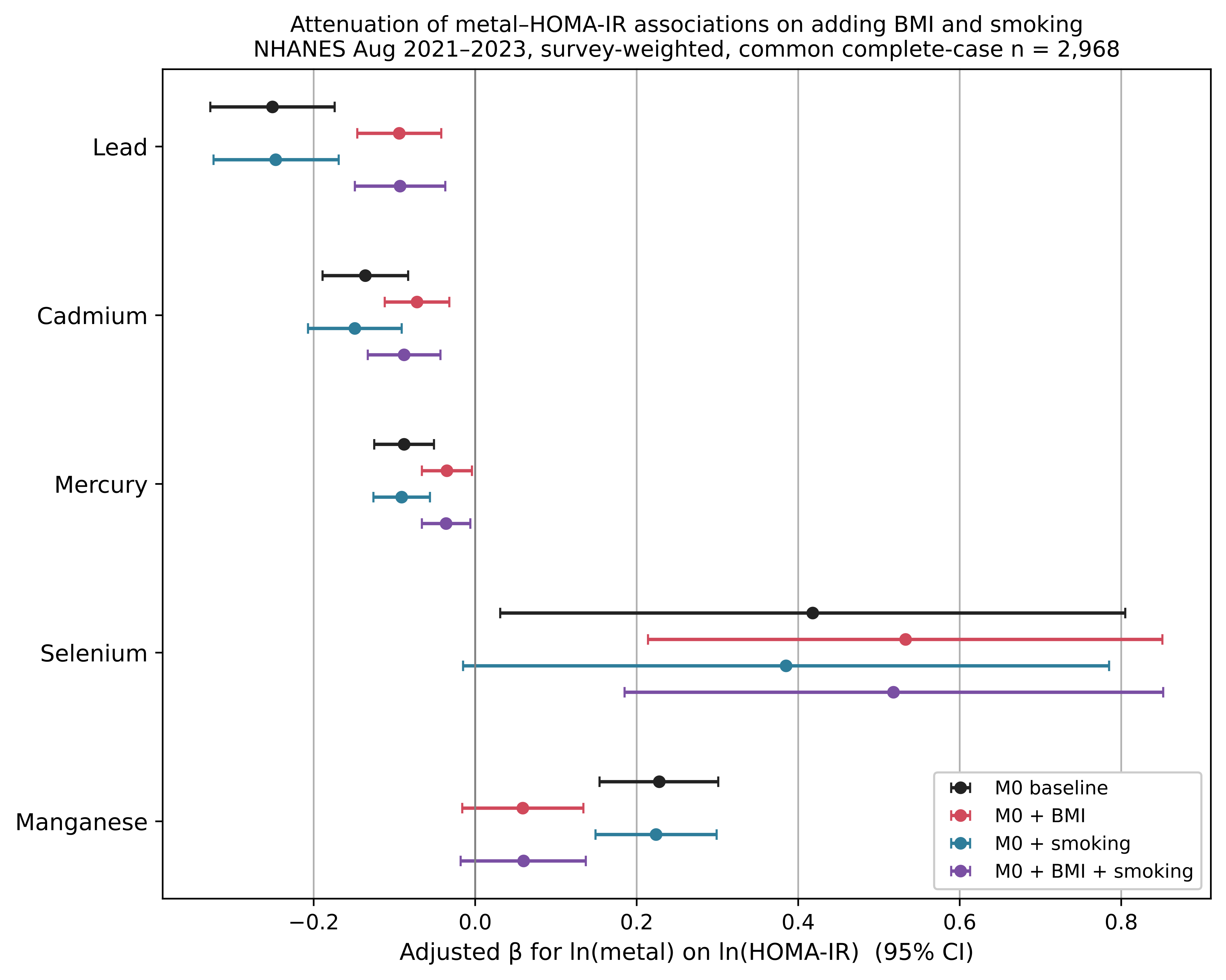


Forest plot of the change in metal–log(HOMA-IR) associations across nested adjustment models (M0 baseline, M1 + BMI, M2 + smoking, M3 + BMI + smoking). Adding BMI attenuates the inverse lead, cadmium, and mercury associations by roughly half to two-thirds and abolishes the positive manganese association, whereas adding smoking alone changes the estimates negligibly. Bars show 95% confidence intervals computed on the survey design degrees of freedom (15).
